# Neuronal mTOR Outposts: Implications for Translation, Signaling, and Plasticity

**DOI:** 10.3389/fncel.2022.853634

**Published:** 2022-04-07

**Authors:** Bekir Altas, Andrea J. Romanowski, Garrett W. Bunce, Alexandros Poulopoulos

**Affiliations:** Department of Pharmacology, University of Maryland School of Medicine, Baltimore, MD, United States

**Keywords:** mTOR, local translation, axon, synapse tagging, ribosome biogenesis, ketamine, axon regeneration

## Abstract

The kinase mTOR is a signaling hub for pathways that regulate cellular growth. In neurons, the subcellular localization of mTOR takes on increased significance. Here, we review findings on the localization of mTOR in axons and offer a perspective on how these may impact our understanding of nervous system development, function, and disease. We propose a model where mTOR accumulates in local foci we term mTOR outposts, which can be found in processes distant from a neuron’s cell body. In this model, pathways that funnel through mTOR are gated by local outposts to spatially select and amplify local signaling. The presence or absence of mTOR outposts in a segment of axon or dendrite may determine whether regional mTOR-dependent signals, such as nutrient and growth factor signaling, register toward neuron-wide responses. In this perspective, we present the emerging evidence for mTOR outposts in neurons, their putative roles as spatial gatekeepers of signaling inputs, and the implications of the mTOR outpost model for neuronal protein synthesis, signal transduction, and synaptic plasticity.

## Introduction

The longest cells in the human body are projection neurons with axons that innervate the toes from the spine. Each one of these neurons has a legs’ worth of uninterrupted plasma membrane and cytoplasm (Bionumbers ID 104901; Cavanagh, 1984; Milo et al., 2010). In development, these cells begin with most of their cytoplasm in the cell body. We can describe their location as centered around their nucleus, the way we describe most cells at the anatomical scale. Soon thereafter, they incorporate biomass and increase in length by a factor of tens of thousands (Cleveland, 1996). At the end of development, while the neuron’s nucleus remains in the spinal cord, most of the rest of the neuron is more so located in the leg. So too are most of the sites of cellular growth, which are macroscopically distant from the cell’s central command in the nucleus. How does cellular machinery coordinate such intense growth at a distance? How is the nucleus involved in local growth processes, like axon projection and synaptic plasticity, with selectivity across such expansive cellular real estate? We propose here that neurons have developed peculiar features in their mechanisms for controlling cellular growth, centered around the kinase mTOR, as potential solutions to these unique problems.

mTOR is a key regulator of growth in eukaryotic cells from yeast to neurons. Sossin and colleagues appropriately call it an “anabolic autocrat” (Graber et al., 2013), as it appears to control every pathway regulating the synthesis of biomass from nutrients and its breakdown back into nutrients. mTOR receives input about the availability of building-blocks (e.g., amino acids and glucose), energy (e.g., ATP), and oxygen. In multi-cellular organisms, mTOR additionally transduces signals from growth factor receptor pathways that mediate trophic signaling in development and adulthood to coordinate multi-cellular growth and homeostasis (Saxton and Sabatini, 2017).

In the brain, mTOR-dependent functions are particularly diverse (Hoeffer and Klann, 2010), expanding beyond canonical growth to the consolidation of memory (Graber et al., 2013), the regeneration of injured axons (Lu et al., 2014), and even the action of antidepressants (Li et al., 2010). Beyond the well-appreciated roles of mTOR in cancer, an emerging spectrum of conditions causing epilepsy and effecting cognition are being recognized as “mTORopathies” (Costa-Mattioli and Monteggia, 2013; Crino, 2016). The striking range of function and dysfunction involving mTOR in the nervous system highlights the importance of understanding mTOR cell biology in native neuronal contexts.

One of the most striking features of mTOR biology is its position as a signaling bottleneck at the center of a dense network of convergent input and divergent output pathways. We propose a model by which neurons take advantage of mTOR’s nodal position across multiple pathways to create a spatial gate for local signaling by focally regulating mTOR’s subcellular localization. We name this the mTOR outpost model, according to which, mTOR complexes accumulate in local outposts at select subcellular sites along neuronal processes. The presence or absence of outposts determines whether the mTOR-dependent branches of local signaling pathways are amplified toward cell-wide responses. In so doing, even spinal cord neurons are able to respond to growth signals as far away as the toe.

Here we review the evidence for local mTOR outposts in neurons and examine the implications of the mTOR outpost model on protein synthesis, signal transduction, and synaptic plasticity. We end on a forward-looking note, discussing the open questions and experimental approaches to challenge and refine the model.

## Subcellular Localization of mTOR and the mTOR Outpost Model

Studies in yeast and cell lines show that mTOR can associate with select subcellular organelles (Betz and Hall, 2013). In these cells, mTOR can colocalize with organelle markers for lysosomes (Abu-Remaileh et al., 2017; Rogala et al., 2019), endosomes (Flinn et al., 2010; Hatakeyama and De Virgilio, 2019; Bronfman and Moya-Alvarado, 2020), as well as the endoplasmic reticulum (Boulbés et al., 2011; Ebner et al., 2017) and focal adhesions (Rabanal-Ruiz et al., 2021). In neurons, mTOR is generally thought to mirror these basic patterns, and to associate with postsynaptic sites in dendrites, where mTOR-dependent signaling functions in synaptic plasticity (Costa-Mattioli and Monteggia, 2013; Sossin and Costa-Mattioli, 2019).

**Figure 1:**
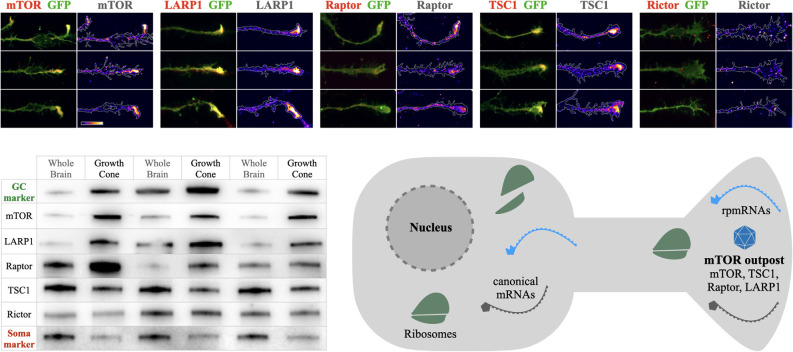
mTOR outposts and the mTOR outpost model. Top panels: examples of mTOR outposts in axon growth cones of cultured cortical neurons seen as dense accumulations of mTOR, LARP1, Raptor, and TSC1, but not of Raptor. Green shows membrane-anchored GFP outlining the axon growth cone. Immunoreactivity of each protein is overlaid in red and shown alone in density heat maps. Scale bar is 10 μm and shows heat map color-range from lowest (left) to highest (right) intensity. Bottom left: Western blots of newborn mouse brain lysates and growth cone fractions showing enrichments of mTOR and associated proteins imaged above. Growth cone marker GAP43 (GC marker, green) and cell body marker GM130 (Soma marker, red) shown for reference. Three independent subcellular fractionation experiments are shown, with whole brain lysates in odd lanes and growth cone fractions in even lanes. mTOR, LARP1, and Raptor are highly enriched in growth cones and depleted from soma-rich lysate, while TSC1 and Rictor are present, but not enriched in growth cones. Data panels are adapted from Macklis and colleagues (Poulopoulos et al., 2019). Bottom right: schema of the mTOR outpost model in a neuron. Local densities of mTOR and associated proteins, including Raptor (key subunit of mTORC1), TSC1 (master regulator of mTOR activation), and LARP1 (mTORC1 and TOP mRNA binding protein), accumulate in local densities into distal processes forming local mTOR outposts (shown as blue polyhedron). Ribosomal protein mRNA transcripts (rpmRNAs, light blue) with 5’ TOP motif that interact with LARP1 and mTORC1 for translation, are enriched in compartments together with mTOR outposts.

Recent evidence for mTOR in axons has emerged. In developing projection neurons, mTOR is observed to accumulate in dense local foci at the tips of growing axons in dorsal root ganglion neurons of the PNS (Terenzio et al., 2018) and in cortical projection neurons of the CNS (Poulopoulos et al., 2019). While their subcellular structure and molecular content have yet to be fully determined, these mTOR-dense axon foci are enriched in key mTOR-associated proteins, including TSC1, Raptor, and Larp1 (Figure 1). We propose naming these structures “mTOR outposts” [analogous to the term “Golgi outposts” (Horton et al., 2005)], representing local signaling platforms that accumulate neuronal mTOR and localize it to strategic sites within neurons.

Many open questions remain regarding the content and dynamics of mTOR outposts. Are they a feature specific to development, or do they perdure in adulthood? Are they specific to axons, or do they appear in the somatodendritic compartment as well? Are they stable structures that traffic across neuronal processes, or do they form *in situ* by local translation of mTOR as seen in the PNS (Terenzio et al., 2018)? What is the structural substrate of mTOR outposts? Outposts in axon growth cones are found together with mRNA and RNA-binding proteins (Poulopoulos et al., 2019), which may relate to mTOR seen in RNA-containing phase condensates, such as p-bodies and stress granules (Takahara and Maeda, 2012; Wippich et al., 2013; Zhang et al., 2018). Do outpost carry mTOR Complex 1 (mTORC1) or 2? The mTOR outposts seen in axon growth cones of cortical neurons contain Raptor but not Rictor (Figure 1), indicating these are specifically mTORC1 outposts. While mTORC2 outposts have not yet been observed, subcellular localization of mTORC2 has been suggested to spatially regulate signaling (Ebner et al., 2017). Pending further investigation, we propose a general mTOR outpost model, with known mTORC1 outposts in developing cortical axons, and putative mTORC2 outposts in other contexts, until the molecular content of outposts is further studied across cellular compartments, neuronal subtypes, and developmental stages.

We postulate here that mTOR outposts are a generalizable feature of neuronal cell biology. We hypothesize their presence and function in axons beyond development, as well as a role for synaptic outposts in dendritic or axonal compartments in adulthood. Processes known to critically implicate mTOR in the adult brain include structural plasticity in long-term memory (Costa-Mattioli and Monteggia, 2013; Graber et al., 2013) and axon regeneration (Abe et al., 2010; Lu et al., 2014), both involving sprouting and growth of neuronal processes. It is appealing to consider that mTOR outposts have a role in sprouting and growth, which occurs *en masse* during development, and selectively in adulthood through transient reinstatement of the developmental state for local circuit remodeling.

The many roles of mTOR in circuit development, plasticity, and regeneration suggest that neuronal mTOR is tuned to function within the macroscopic scales of nervous system cell biology. Below, we explore some of the implications on these processes of the mTOR outpost model in the developing and adult brain.

### mTOR Outpost Model in Regulating Neuronal Protein Synthesis

A key function of mTOR signaling is regulating the intensity of protein synthesis (Thoreen, 2017). Activation of mTOR causes mild increases in cap-dependent translation for most canonical transcripts. Yet a small set of non-canonical transcripts exhibit a much more dramatic all-or-none dependence on mTOR for translation. These are the ~80 ribosomal protein mRNAs (rpmRNAs) together with a handful of other proteins that make up the translation machinery of the cell. All-or-none regulation by mTOR is achieved through a sequence on rpmRNAs called the 5’TerminalOligoPyrimidine (TOP) motif (Meyuhas and Kahan, 2015). mTOR complexes bind TOP motifs through LARP1, an auxiliary RNA-binding protein of mTORC1 (Tcherkezian et al., 2014; Fonseca et al., 2015), which is enriched in axon mTOR outposts together with rpmRNAs (Poulopoulos et al., 2019). Production of new ribosomal proteins from rpmRNAs is blocked until mTORC1 interacts with and phosphorylates LARP1 bound to the TOP motif (Thoreen et al., 2012; Tcherkezian et al., 2014; Fonseca et al., 2015). mTOR additionally controls the production of rRNA (Mayer and Grummt, 2006), and as such tightly controls ribosome biogenesis and the total ribosomal content of the cell. This enables mTOR to determine cell-wide rates of protein synthesis based on the inputs it receives from nutrient availability and growth factor signaling.

**Figure 2:**
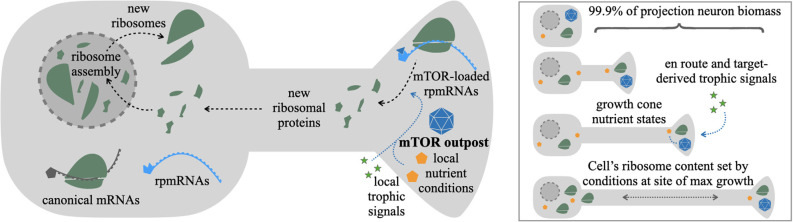
mTOR outpost model in the regulation of ribosome content. New ribosomes are assembled in the nucleus from three rRNAs produced in nucleoli, and ~80 ribosomal proteins imported from the cytosol, where each is translated from ribosomal protein mRNAs (rpmRNAs). rpmRNAs are consistently found enriched in distal processes of neurons. Unlike most canonical mRNAs (gray), rpmRNAs (cyan) have an atypical 5’ TOP motif that requires direct binding and activation by mTOR complexes (blue triangle) to initiate translation. The mTOR outpost model predicts that rpmRNA-rich processes with localized mTOR outposts (blue polyhedron) would be a major source of new ribosomal proteins (green). The rate of ribosomal protein production would depend on local growth-stimulating conditions at the mTOR outpost, such as nutrient availability (yellow pentagons) and local trophic signals (green stars). As a result, in developing projection neurons with mTOR outposts and rpmRNAs in distal axon growth cones, the rate of cell-wide ribosome production would be determined by target-derived trophic signals and the local metabolic conditions at the axon growth cone, the sites of growth for the majority of a projection neuron’s biomass.

The mTOR outpost model predicts specific effects on the regulation of a neuron’s ribosomal content. Rather than integrate growth conditions evenly across the cell, the model predicts that local conditions at sites containing mTOR outposts will weigh more heavily toward regulating ribosome production than conditions in other parts of the cell. For example, axon growth cones are enriched in mTOR and rpmRNAs several fold over the cell body (Figure 1; Poulopoulos et al., 2019). This organization suggests that signals and nutrient conditions at growth cones may have more influence on the overall rates of ribosome production in the cell than growth conditions proximal to the cell body. We posit that projection neurons may have adapted this mechanism to regulate cell-wide ribosome content by localizing mTOR outposts and rpmRNAs to the subcellular sites where the majority of cellular growth occurs (Figure 2).

The localization of rpmRNAs to such distal processes is curious given that the nucleus is the canonical site of new ribosome assembly. Nonetheless, rpmRNAs have consistently been found enriched in distal neurites in a number of studies (Holt et al., 2019; Perez et al., 2021). Indeed, an alternative hypothesis to explain the presence of some rpmRNAs in the distal processes postulates the refurbishment of existing ribosomes with new protein through local translation of rpmRNAs. Supporting this hypothesis are two recent studies. The first by Holt and colleagues provides direct evidence from frog retinal axons that a subset of rpmRNAs encoding superficial ribosomal proteins are translated in axons and their products are locally incorporated onto existing axon ribosomes (Shigeoka et al., 2019). The second study by Schuman and colleagues (Fusco et al., 2021) found 70 rpmRNAs actively translating in the neuropile strata of the mouse hippocampus. Of these, 12 ribosomal proteins corresponding to superficial proteins on the ribosome, were termed “exchangers” because they rapidly incorporate onto ribosomes even after ribosome biogenesis from the nucleus was pharmacologically inhibited. These elegant experiments provide strong evidence for the refurbishment model of superficial ribosomal proteins locally replacing subunits on existing ribosomes in distal processes, providing a less circuitous and energetically more favorable interpretation for the distal pool of rpmRNAs.

Beyond local ribosome refurbishment, a number of rpmRNAs encoding core ribosomal proteins that do not locally incorporate into existing ribosomes are still enriched in distal processes (Poulopoulos et al., 2019; Perez et al., 2021). We propose that this broad localization of rpmRNAs can be understood through the mTOR outpost model: rpmRNAs localize with distal mTOR outposts in order to centrally regulate new ribosome production based on the local growth conditions at the sites of most intense cellular growth. This would serve as an adaptive feature for neurons to scale their protein producing capacity according to the needs of cellular growth. From an evolutionary perspective, this interpretation: (1) maintains the strongly conserved mechanism of ribosome biogenesis in the nucleus, with its important quality control steps that rely on nuclear export (Henras et al., 2008); and (2) provides an evolutionary intermediate that facilitates the emergence of the local ribosome refurbishment mechanism. Once mechanisms to localize rpmRNAs to distal processes have been established to serve mTOR outpost local regulation, local translation and integration of those subunits that benefit local refurbishment of ribosomes can easily emerge.

The high levels of shuttling between nucleus and axons required for distal rpmRNA translation and nuclear ribosome assembly beg the question, how would developing neurons benefit from such circuitous transport cycles? One interpretation of why localizing rpmRNA to axons may have benefits that outweigh the burden of distant trafficking becomes apparent when looking at the numbers. Ribosome constituents account for over half of all transcription in yeast (Warner, 1999), and over one-in-10 of every cellular protein in humans (An and Harper, 2020). In developing cortical neurons, ribosome content increases 2–3 fold within the first postnatal weeks (Slomnicki et al., 2016). Cortical projection neurons can have over 99.9% of their final biomass incorporated into their axons (Cleveland, 1996). These numbers show that the cellular economy of developing projection neurons is dominated by two linked processes: ribosome biogenesis and axon growth. The localization of mTOR outposts together with rpmRNAs at the tips of growing axons may serve to accurately couple the regulation of these two critical processes.

To achieve growth on the scale seen in projection neurons, the regulatory benefits of harmonizing local axon growth with the cell-wide capacity for protein synthesis may be crucial enough to make up for circuitous rpmRNA and ribosomal protein trafficking. A study from gut epithelial cells identified the subcellular movement of rpmRNAs to the apical membrane facing the gut lumen after a meal (Moor et al., 2017). This mysterious finding in polarized cells of the gut may be an evolutionary echo of the mechanism we posit for neurons: polarized cells localize their rpmRNAs and mTOR outposts to the subcellular sites where nutrient conditions are most relevant for regulating growth and function.

Future experiments to test this hypothesis would involve local manipulation of growth cone mTOR to see whether it disproportionately impacts the rate of cell-wide ribosome biogenesis, as the model would predict. Direct observation of retrograde transport of nascent ribosomal proteins from distal processes back into the nucleus for incorporation into nascent ribosomes would also need to be seen. Interestingly, of the 70 rpmRNAs actively translated in the hippocampal neuropile, most of them (those not in the subset of 12 “exchangers”) are no longer incorporated into ribosomes after inhibition of nuclear export (Fusco et al., 2021), suggesting that a large fraction of distal rpmRNAs may provide protein for canonical ribosome biogenesis in the nucleus.

### mTOR Outpost Model in Signal Transduction to the Nucleus

The process of selecting which of the many local signals on a neuron’s surface will be transduced to the nucleus is a central conundrum of neuronal signaling. Among the many proposed mechanisms is the retrograde transport of signaling endosomes that physically bring the signal closer to the nucleus (Harrington and Ginty, 2013; Cosker and Segal, 2014). Briefly, local extracellular ligands engaging surface receptors are internalized forming endosomes containing ligand-receptor complexes. These organelles mature into signaling-able late endosomes and are transported toward the cell body where their signal is transduced to the nucleus. The involvement of signaling endosomes in neurons is well documented in the context of trophic factors like NGF (Grimes et al., 1996) and BDNF (Olenick et al., 2019; Bronfman and Moya-Alvarado, 2020).

**Figure 3:**
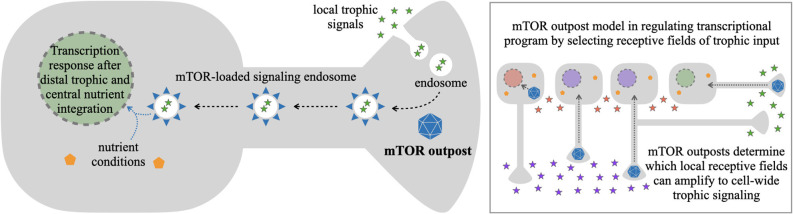
mTOR outpost model in gating local trophic signals reaching the nucleus. Local trophic signals, such as NGF in development and BDNF in adulthood, are dynamically and locally present at different regions of a projection neuron’s plasma membrane. Endocytosis at those regions can lead to endosomes with internalized trophic signals bound to their cognate receptors on the endosome membrane. In those regions that additionally contain a local mTOR outpost (blue polyhedron), mTOR and associated proteins (blue triangles) from the outpost are recruited to local late endosomes leading to putative signaling endosomes with ligand, receptor, and mTOR-quipped transduction apparatus. Signaling endosomes can be transported retrogradely to the cell body, bringing mTOR-dependent trophic signaling to the vicinity of the nucleus. As such, the location of an mTOR outpost would function as a permissive gate to bring distal trophic signals from the periphery towards the regulatory center of the cell, where information on central nutrient conditions could be integrated through activation of mTOR on the signaling endosome. With selective mTOR outpost localization, this would allow neurons to respond transcriptionally to select local or distal signals (various color stars in inset) from select branches of neurites. This would make localization of mTOR outposts in the soma, dendrites, axons, or sub-branches in those compartments, determine critical modes of cellular transcriptional responses to stimuli.

An open question in the signaling endosome model is how are signals selected for transduction to the nucleus, over those which are kept local. The mTOR outpost model provides a putative mechanism for selection through the known interactions of mTOR with late endosomes (Flinn et al., 2010; Hatakeyama and De Virgilio, 2019; Bronfman and Moya-Alvarado, 2020). The model proposes that ligand-receptor complexes are internalized at various sites along the neuron. At sites containing mTOR outposts, these late endosomes will be equipped with mTOR and its associated protein complexes to become mTOR-competent signaling endosomes. Areas without mTOR outposts would retain mTOR-independent signaling, but would not be able to transduce the mTOR-dependent signal. As such, selective localization of mTOR outposts would act as a spatial selectivity filter for which local trophic signals are amplified toward cell-wide responses (Figure 3).

There is strong evidence that mTOR associates with signaling endosomes (Flinn et al., 2010; Hatakeyama and De Virgilio, 2019), including recent direct observation of mTOR activation by axon-derived BDNF-TrkB signaling endosomes (Bronfman and Moya-Alvarado, 2020). Additionally, the combination of mTOR-dependent signaling complexes together with elements of the lysosome in late endosomes provides a potential platform for signal integration between the trophic signaling branch of mTOR (through PI3K and ERK) and the amino acid sensing branch of mTOR (through Rheb). This signal integration *via* mTOR activation on the signaling endosome could combine trophic signals from distal outposts with central nutrient states as the signaling endosome reaches the cell body.

The mTOR outpost model in signal transduction to the nucleus permits a critical feature of neural circuit development: neighboring neurons can respond to distinct extracellular cues, even though their cell bodies are “bathed” in the same extracellular environment. By sequestering cellular mTOR away from the proximal cell body (Figure 1), and localizing it to distal outposts, neurons would be able to attenuate proximal signals from around their cell bodies in order to respond to distal target-derived signals from their outposts (Figure 3 inset). For example, in the developing cortex where mTOR outposts are in axon growth cones, this mechanism would allow developing layer V callosal projection neurons to respond when their axons encounter target-derived NGF in the corpus callosum while neighboring layer V corticospinal projection neurons could independently respond when their axons encounter trophic signals along the pyramidal tract. As such, developing projection neuron subtypes in the same brain region can respond independently to their target-derived signals based on the developmental progression of their growing axons. This would facilitate the execution of projection-specific developmental programs (Greig et al., 2013) even in neurons that share the same ambient growth factor environment around their cell bodies. An equivalent mechanism could manifest in the adult brain in the transduction of select synaptic signals toward the nucleus to elicit long-term structural plasticity, which we examine below.

### mTOR Outpost Model in Synaptic Plasticity and Long-Term Memory

Synaptic activity can result in enduring changes of synaptic weight only when the synthesis of new proteins is permitted. The acute arrest of translation prevents long-term plasticity as well as its behavioral cognate, long-term memory, as shown by protein synthesis inhibitors inducing amnesia (Sossin and Costa-Mattioli, 2019). As a central regulator of protein synthesis, it is no surprise that a wide range of long-term plasticity is mTOR-dependent, often involving BDNF-TrkB signaling (Costa-Mattioli and Monteggia, 2013; Graber et al., 2013). The implications of the mTOR outpost model on the selective distribution of mTOR may thus have consequential implications for which synapses are able to undergo long-term plasticity.

The role of mTOR in synaptic plasticity is largely ascribed to the postsynaptic compartment through mTOR-dependent local translation of dendritic transcripts (Tang and Schuman, 2002; Costa-Mattioli et al., 2009). If dendritic mTOR outposts are observed, the known functions of postsynaptic mTOR can be revisited from the perspective of spatial selectivity introduced by the TOR outpost model. Alternatively, dendritic mTOR may function without spatial selectivity *via* broad distribution throughout the dendritic arbor. Future experiments using sparse labeling knockin methods to track the subcellular patterns of endogenous mTOR throughout a neuron’s compartments will elucidate whether dendritic mTOR also forms outposts.

On the other side of the synaptic cleft, there are few, but well documented, examples of presynaptic mTOR-dependent protein synthesis and synaptic plasticity (Younts et al., 2016). The recent findings of mTOR outposts in developing CNS axons (Poulopoulos et al., 2019), of locally translated mTOR in regenerating PNS axons (Terenzio et al., 2018), and of ubiquitous protein synthesis machinery in presynaptic terminals (Hafner et al., 2019) reinforces the emerging notion of presynaptic components in the mTOR-dependent mechanisms of long-term plasticity and the formation of new memories (Sossin and Costa-Mattioli, 2019).

**Figure 4:**
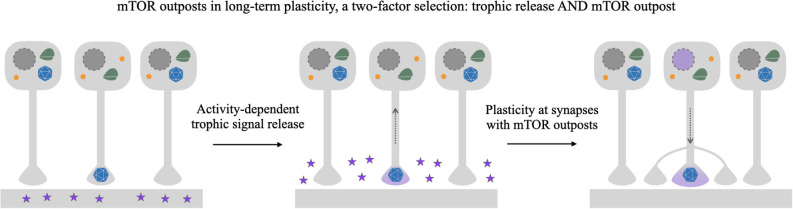
mTOR outpost model in gating long-term plasticity. Schema depicting selective strengthening of connections *via* target-derived trophic signaling and selective localization of putative presynaptic mTOR outposts. In this example, three neurons synapse onto the same segment of dendrite on a postsynaptic neuron. Of the presynaptic cells, only one has an mTOR outpost (blue polyhedron) at the presynaptic terminal, while the other two have somatodendritically localized mTOR. Potentiating synaptic activity causes the postsynaptic neuron to release trophic factors such as BDNF (purple stars) in the vicinity of the presynaptic terminals. While all terminals may have receptors, only terminals containing a presynaptic mTOR outpost will be able to transduce mTOR-dependent signaling to the nucleus, resulting in selective nuclear response (purple nucleus) to activity-dependent target-derived signaling. mTOR-dependent structural plasticity will selectively occur only on contacting presynaptic cells with an axon-localized mTOR outpost, a selection criterion gating plasticity that can be overlaid and combined with direct activity-dependent plasticity mechanisms.

The mTOR outpost model makes several testable predictions about the role that presynaptic mTOR may play in long-term plasticity. When a segment of the dendrite is stimulated, the local dendritic release would expose contacting terminals to BDNF (Kuczewski et al., 2009). The outpost model predicts that not all presynaptic contacts on that dendrite would contain mTOR outposts to respond to the postsynaptic trophic signal. Rather, only those axons that contain mTOR outposts would respond to the mTOR-dependent signal and convey the trophic signaling back toward the cell body of the presynaptic cell triggering the cell-wide responses required for long-term plasticity (Figure 4).

The responses elicited by trophic signals in terminals containing mTOR outposts may include both local effects of mTOR signaling, such as cytoskeleton remodeling and local translation, as well as cell-wide effects inducing transcription and translation. For the latter, a mechanism such as mTOR-loaded signaling endosomes presented above could convey the local trophic signal toward the nucleus. In turn, the transcriptional products of the nuclear response may be captured by mTOR outpost synapses through their local mTOR-dependent effects. Those could enable the growth of existing synapses, as well as induce synapse sprouting of new contacts through growth cones. Studies in Aplysia demonstrate that local inhibition of mTOR at presynaptic terminals prevents synapse growth associated with long-term plasticity (Casadio et al., 1999). In these classic Aplysia experiments, there is an interesting interplay between terminal-to-terminal and terminal-to-nucleus signaling, together with the localized application of rapamycin, which may offer an elegant experimental system to study the mTOR outpost model. Further investigation of the putative role of mTOR outposts in synaptic plasticity would require the development of local outpost-specific manipulation of mTOR signaling. Local pharmacological approaches are poorly suited to discriminate between presynaptic and postsynaptic mTOR. The poor resolution of local pharmacological infusion hampers investigations of local activation or inhibition at individual synapses along a segment of dendrite. Future approaches using photo-activatable and local ablation approaches with high spatial specificity will enable investigations of the putative role of mTOR outposts in providing an added layer of selectivity to signals promoting long-term plasticity.

The predictions the model makes of a privileged subset of synapses defined by a molecular identifier, the mTOR outpost, are very much in line with those hypothesized by inclusive theories of long-term memory, including the Synapse Tagging and Capture model (Redondo and Morris, 2011) and the Memory Synapse model (Sossin, 2018). These models propose solutions for how long-term plasticity achieves synapse-specificity through cell-wide responses. Both models posit hypothetical molecular identifiers at select synapses able to respond to potentiation. In the Synapse Tagging and Capture model, the products of the cell-wide transcriptional and translational response are specifically captured by tagged synapses to strengthen the connection. The consequent strengthening of synaptic weights in both models involves the incorporation of newly synthesized protein to existing synapses and the formation of new synapses.

The presence or absence of mTOR outposts offers a candidate molecular identifier of tagged synapses or memory synapses. The mTOR outpost provides a gating mechanism for signal transduction to the nucleus to elicit cell-wide responses to potentiating stimuli. It additionally provides a conceptual mechanism for tagging synapses to capture stimulated products through local mTOR-dependent effects, e.g., local cytoskeletal effects. Finally, the requirement of mTOR for the growth of cortical axons (Poulopoulos et al., 2019) and the induction of adult axon sprouting by mTOR activation after injury (Abe et al., 2010; Lu et al., 2014) suggest that axon mTOR outposts may be central to axon growth. They may represent abroad feature in development that is selectively reinstated in adulthood for local axon sprouting and new synapse formation as required for long-term memory. We look forward to targeted investigations to directly test whether mTOR outposts may serve as synapse tags in models of long-term memory.

Two key sets of questions emerge: (1) do mTOR outposts occur in synapses, and if so, are they common or rare? In other words, what is the selectivity value of an mTOR outpost? and (2) how is the localization of mTOR outposts at the synapse determined? Is there a dendritic mode vs. an axonal mode of localization? Are specific branches of axons or dendrites selected? Does activity instruct outpost localization? These questions remain wide open and will require the development of capabilities to follow the behavior of native mTOR outposts within intact synaptic circuits. Currently, the only evidence for how local mTOR outposts may arise comes from experiments in regenerating PNS axons where mTOR transcripts are shown to localize and locally translate and accumulate mTOR (Terenzio et al., 2018). The dynamics and localization of mTOR outposts may thus be an important feature for axon growth broadly, and maybe related to the inability of CNS axons to natively regenerate in adults. Interestingly, exogenous activation of mTOR reinstates axon growth in adult CNS (Lu et al., 2014). Investigations into mTOR outposts and their putative roles in long-term plasticity and CNS regeneration are fascinating prospects.

### mTOR Outpost Model in Pharmacology: The Case of Fast-Acting Antidepressants

The mTOR outpost model may offer alternative interpretations in experiments using local pharmacological inhibition of mTOR with common agents like Rapamycin and Torin. For example, mTOR signaling has been extensively investigated in the pathogenesis and treatment of depression (Abelaira et al., 2014; Duman et al., 2016; Fukumoto et al., 2019). Fast-acting antidepressants, such as scopolamine and ketamine (Gould et al., 2019), lose their efficacy when mTOR is inhibited by local rapamycin infusion into the rodent prefrontal cortex (PFC; Li et al., 2010, 2011; Voleti et al., 2013; Fukumoto et al., 2019). These results have largely been interpreted as showing mTOR-dependence of fast-acting antidepressants. However, in other experimental paradigms of ketamine’s antidepressant action, mTOR-dependence is not observed. Unlike local infusion to the PFC, systemic delivery of rapamycin does not inhibit ketamine efficacy in rodents (Autry et al., 2011). Most importantly, systemic mTOR inhibition appears to prolong rather than abrogate the antidepressant efficacy of ketamine when rapamycin is co-administered to human patients (Abdallah et al., 2020).

The mTOR outpost model introduces the possibility that local PFC infusion of rapamycin may target select axon mTOR outposts belonging to distant projection neurons with afferents to the PFC. This perspective offers alternative interpretations for some of the seemingly disparate datasets. One interpretation is that ketamine acts on postsynaptic neurons in PFC to release BDNF (Autry et al., 2011), which activates presynaptic mTOR in the small fraction of projections to the PFC that contain axon mTOR outposts (similar to Figure 4). Selective plasticity at these PFC synapses with axon mTOR outposts could sculpt the circuit-level changes manifesting ketamine’s antidepressant efficacy. In this view, local rapamycin injections into PFC would block antidepressant efficacy by negating the competitive advantage of the privileged mTOR outpost-containing projections to the PFC, thus making them equal to other outpost-less projections to PFC. This would eliminate an underlying structure in the potential for plasticity and thus block the circuit-sculpting effects required for antidepressant action. Alternatively, select postsynaptic sites with mTOR outposts in PFC neurons receiving afferent contacts would be affected by local rapamycin infusion, and equivalently an underlying structure in the potential for plasticity would be lost.

The predictions of our model are that local inhibition of mTOR outposts can interfere with the essence of the outpost model, namely the underlying patterns of synapses with outposts that have the potential for mTOR-dependent plasticity, vs. those which do not. Conceivably, systemic administration of rapamycin may not interfere with the underlying patterns set by mTOR outpost. Provided it is not in high enough doses for complete and total inhibition of mTOR signaling, systemic Rapamycin would scale mTOR responses evenly across the brain, meaning that privileged synapses containing outposts would still be able to respond to trophic signals more so than their outpost-less neighbors, thus preserving the underlying structure in place for circuit-sculpting plasticity. From this perspective, it is plausible that inducing mTOR outpost-dependent plasticity with ketamine while attenuating its intensity with systemic rapamycin may well achieve a sweet spot that improves and prolongs antidepressant efficacy, as observed in patients where these compounds were co-administered (Abdallah et al., 2020).

Given the therapeutic importance and potential for optimizing pharmaco therapies against depression, research labs at AbbVie, Servier, Pfizer, and Alkermes revisited the question of whether ketamine activates mTOR using PFC synaptosomes. They concluded that “detection of the effects of ketamine on mTOR seem to require special conditions that are difficult to identify and establish,” reporting marginal and variable synaptic activation of mTOR in their hands (Popp et al., 2016). If axon mTOR outposts are the target of Ketamine-induced BDNF as posited above, the observations of marginal mTOR activation in PFC synaptosomes may be reconciled with previous results indicating an mTOR-based mechanism for antidepressant action. In these experiments, mTOR in bulk synaptosomes from PFC would mostly comprise postsynaptic mTOR from neurons residing in the PFC, with only a minor contribution coming from the biologically relevant axon mTOR outposts from select PFC afferents. This minor fraction of mTOR activation, though therapeutically relevant, would appear as a marginal signal in a mix of bulk PFC synaptosomes.

There are other cases where experiments on mTOR could yield apparent discrepancies if mTOR outposts are not considered. For example, genetic inhibition of mTOR (by conditional knockout or expression of mTOR suppressor genes) in a brain area would leave intact axon mTOR outposts from afferents with cell bodies outside that area. This would create discrepancies in the interpretations of pharmacological vs. genetic experiments that, without considering the mTOR outpost model, would seem to target the same pathway in the same brain region with conflicting results. Similar discrepancies may arise when comparing *in situ* or RNAseq data to immunolabeling or proteomic data from the same brain areas. The mTOR outpost model posits that the transcript source of mTOR in the cell body may be macroscopically distant from the functionally important protein source in distal outposts.

In these examples, experiments need not be interpreted through a binary lens of whether a certain process is mTOR-dependent or not. Rather, the model offers more refined interpretations based on mTOR outpost localization and synapse-specificities, which may underly the structure of plasticity even before plasticity is elicited. In this and other such cases, reinterpretation of prior data from the perspective of the mTOR outpost model may rectify previous inconsistencies and add to our mechanistic insight with potential therapeutic implications.

## Discussion

The mTOR outpost model we propose, if confirmed, has many implications across cellular and systems neuroscience. As such, in closing, we focus on thoughts on interrogating the model.

A key challenge of investigating native subcellular mTOR distributions and interrogating the mTOR outpost model is the ubiquitous expression of mTOR in the brain. Labeling endogenous mTOR in dense neuropile with many cellular sources makes identification of subcellular foci within individual axons and dendrites difficult. Exogenous expression approaches, like plasmid or viral overexpression, enable sparse labeling in individual cells. However, overexpression floods the cell with mTOR, masking the selectivity of its endogenous subcellular targeting. Alternative labeling approaches will be necessary to preserve endogenous mTOR localization while achieving single-cell labeling to directly visualize the behavior of mTOR outposts. Approaches such as fluorescent intracellular nanobodies (Gross et al., 2013) or sparse *in vivo* knockin (Mikuni et al., 2016; Richardson et al., 2020) have the potential to overcome these limitations.

With the development of new tools to visualize native mTOR outposts and their dynamics, key questions such as those we highlight above can begin to be mechanistically interrogated *in vivo*. These future investigations will be instrumental to our understanding across the range of mTOR-dependent processes in the brain. We look forward to investigator teams challenging and refining the mTOR outpost model to uncover its roles in the developing, adult, and regenerating nervous system.

## Data Availability Statement

The original contributions presented in the study are included in the article, further inquiries can be directed to the corresponding author.

## Author Contributions

All authors listed have made substantial intellectual contributions to the ideas and writing of this manuscript, and approved it for publication.

## Conflict of Interest

The authors declare that the research was conducted in the absence of any commercial or financial relationships that could be construed as a potential conflict of interest.

## Publisher’s Note

All claims expressed in this article are solely those of the authors and do not necessarily represent those of their affiliated organizations, or those of the publisher, the editors and the reviewers. Any product that may be evaluated in this article, or claim that may be made by its manufacturer, is not guaranteed or endorsed by the publisher.
